# Mortality, violence and access to care in two districts of Port-au-Prince, Haiti

**DOI:** 10.1186/1752-1505-3-4

**Published:** 2009-03-24

**Authors:** Frédérique Ponsar, Nathan Ford, Michel Van Herp, Silvia Mancini, Catherine Bachy

**Affiliations:** 1 Médecins Sans Frontières, 94 rue Dupré, Brussels, Belgium; 2 Médecins Sans Frontières, 49 Jorissen Street, Johannesburg 2017, South Africa; 3 Faculty of Health Sciences, Simon Fraser, University, Vancouver, Canada

## Abstract

**Background:**

Towards the end of 2006 open conflict broke out between United Nations forces and armed militia in Port-au-Prince, Haiti. Fighting was most intense in the district of Cité Soleil.

**Methods:**

A cross-sectional, random-sample survey among the conflict-affected populations living in Cité Soleil and Martissant was carried out over a 4-week period in 2006 using a semi-structured questionnaire to assess exposure to violence and access to health care. Household heads from 945 households (corresponding to 4,763 people) in Cité Soleil and 1,800 household (9,539 people) in Martissant provided information on household members. The average recall period was 579 days for Cité Soleil and 601 days for Martissant.

**Results:**

In Cité Soleil 120 deaths (21 children) were reported (CMR 0.4 deaths/10,000 people/day; <5 MR 0.5 deaths/10,000/day) while in Martissant 165 deaths (8 children) were reported (CMR 0.3/10,000 people/day; <5 MR 0.2/10,000 people/day). Violence was reported as the main cause of adult mortality in both locations (mainly gunshot wounds) accounting for 29.2% of deaths in Cité Soleil and 23% of deaths in Martissant. 22.9% of families in Cité Soleil and 18.6% in Martissant reported at least one victim of violence. Destruction of property and belongings was common in both Cité Soleil (52.4% of families) and Martissant (14.9%). Access to health services was limited, with 11% (22/196) of victims of violence in Cité Soleil and 23% (49/212) in Martissant unable to access care due to insecurity or lack of money.

**Discussion:**

Extrapolating to the total population of these two districts some 2,000 violent deaths occurred over the recall period. Among the survivors, violence had lasting effects in terms of physical and mental health and loss of property and possessions.

## Background

Haiti is one of the poorest countries in the Northern hemisphere, with more than half its 8.5 million population living on less than $US1 per day [1]. The country has been ravaged by political violence for most of its history. The most recent wave of violence broke out in February 2004, following an armed insurrection that overthrew Jean-Bertrand Aristide, then president of Haiti. French and American forces, mandated by the United Nations (UN), arrived in the capital to maintain security; these were replaced several months later by UN stabilization forces. From October 2004, clashes between the police and partisans of president Aristide erupted in several poorer districts of the capital.

Violence and insecurity continued the following year, affecting several neighborhoods in Port-au-Prince and spreading to other towns in the country until elections were held in February 2006. During this period – from the departure of President Jean-Bertrand Aristide to the end of 2005 – an estimated 8,000 people were murdered and 35,000 women sexually assaulted [2].

While the electoral period provided some respite, sporadic incidents of violence continued throughout 2006. Towards the end of that year open conflict broke out between UN forces and armed groups. Fighting was most intense in the Cité Soleil district, considered to be the stronghold of armed militia supporting ex-president Aristide. UN peacekeeping operations intensified in Cité Soleil from the end of 2006 to February 2007, resulting in a drop in criminal violence. However, in other districts in Port-au-Prince, criminal violence continued.

Médecins Sans Frontières (MSF) has been providing medical assistance in different areas of Haiti since 1991. In mid-2007 MSF carried out epidemiological surveys in two districts of Port-au-Prince (Cité Soleil and Martissant) to assess exposure to violence and access to health care for civilian victims of violence. MSF was treating victims of violence in health structures in these two districts, but no assessment of the level of violence in the community had previously been carried out. This article presents the main findings of these surveys.

## Methods

We carried out cross-sectional surveys in Cité Soleil (31 July – 7 August 2007) and Martissant (21 – 31 August 2007) to assess causes of mortality, level and type of violence, and access to health care services. The survey covered the period 1 January 2006 to the end of the survey (average recall period 579 days for Cité Soleil and 601 days for Martissant).

### Study setting

Cité Soleil (200,000 inhabitants) is one of the poorest districts of Port-au-Prince. The urban warfare that followed Aristide's departure in 2004 cut off the population from the rest of the town. Between August 2005 and December 2007 MSF worked in two state-run health facilities located in the slum of Cité Soleil. Martissant (165,000 inhabitants) is a densely populated district to the south of Port-au-Prince. Armed groups supported by different political parties dominated the district, which was largely devoid of government control during the conflict. At the end of 2006 MSF opened an emergency centre providing stabilization and referral services for trauma, obstetric, and surgical emergencies. In July 2007, mobile clinics were established, offering primary health care across Martissant.

### Sampling

We did simple random sampling in Cité Soleil. For an assumed 2% mortality per year in Cité Soleil, an average household size of 5, and a precision of ± 0.4%, we calculated that 945 households needed to be surveyed; this was increased by 20% given that a substantial number of houses in Cité Soleil were known to be abandoned. In total, 1,133 households were included. Survey teams were instructed to note all abandoned buildings and based on this the sample size was recalculated at the end of the survey proportional to the population living in each sub-district. The total sample kept for analysis included 945 households (corresponding to 4,763 people), with the initial level of precision maintained. For the selection of households, we used a satellite map of Cité Soleil [3] that could identify all buildings of the Cité by sub-district. The sample was divided into sub-districts in proportion to the number of buildings in each sub-district. Buildings were numbered and randomly selected using a list of randomly generated numbers by EPI INFO (version 6.04). Each team of surveyors had a map marking all the buildings to be surveyed. If several households lived in one building, one was randomly selected using a random number table.

In Martissant 1,800 families (9,539 people) were surveyed using a two-stage cluster random sampling method (simple random sampling by aerial mapping was not possible because uneven terrain and heavy vegetation prevented identification of all dwellings). Sample size was calculated using the same assumed mortality per year (2%) and precision (± 0.4%). Given an average size of household of 5.25 and a cluster effect of 2, the total sample required was 1,800 households. 200 clusters of 9 families were selected to ensure broad sampling and minimize cluster effect; these were divided into sub-sections proportional to population size. The start of each cluster in each sub-section was randomly selected using map co-ordinates and survey teams proceeded to the nearest house on the right until completion of the cluster.

### Survey questionnaire

We used a semi-structured questionnaire adapted from surveys used in other conflict settings [4]. This was translated from French to Creole, back translated by a different translator, and piloted in Cité Soleil in an area not selected for inclusion in the survey. Questions related to mortality were directed at heads of household, while questions related to violence were directed at those affected when present and consenting (children <15 years were excluded); otherwise the head of household was interviewed. Survey teams were recruited from the community in Cité Soleil (six teams of two interviewers, including 2 women) and Martissant (10 teams of two interviewers, including 7 women), and overseen by 2 supervisors. Data entry was checked on a daily basis by supervisors and as an additional control 5% of forms were randomly checked by the survey co-ordinator.

### Human subject protection

In each selected household, surveyors explained to the head of household the purpose of the study and that confidentially and anonymity would be protected. Children <15 years were excluded from questions relating to violence; if they were affected, questions relating to their experience were directed at heads of household instead of the victims. Oral consent was sought and if refused the team proceeded to the next nearest house (there were 8 refusals in Cité Soleil and 72 in Martissant). Teams were trained to insist on full confidentiality of all information gathered and to explain the medical role of MSF and the objectives of the survey so that people would know that they would not be at risk by sharing information. An MSF ambulance was available in case participants with severe medical conditions were encountered. In case of non-urgent needs, patients were encouraged to seek care at the MSF-supported structures, which provide free care. Participants did not receive any material compensation.

### Statistical analysis

Data were analysed using EPI INFO-6.04 (CDC, Atlanta). For each point estimate for Martissant the design effect was estimated in CSample (EpiInfo) to obtain 95% confidence intervals.

## Results

Survey results are presented according to the direct (mortality, physical and psychological harm) and indirect (displacement and destruction of property and possessions) consequences of violence.

### Mortality

In Cité Soleil, 120 deaths were reported for the period studied, of which 21 were among children <5. This corresponds to a crude mortality rate of 0.4/10,000/day (95%CI: 0.4–0.5), and an <5 mortality rate of 0.5/10,000/day (95%CI: 0.3–0.7) (Table 1). In Martissant, 165 deaths were reported, of which 8 were among children <5 years, corresponding to a crude mortality rate of 0.3/10,000 people/day (95%CI: 0.2–0.3) and an <5 mortality rate of 0.2/10,000 people/day (95%CI: 0.1–0.3).

**Table 1:** Mortality in Cité Soleil and Martissant

	**Cité Soleil**	**Martissant**
***Mortality rate***		
CMR	0.4 [0.4–0.5*]	0.3 [0.2–0.3]
<5 MR	0.5 [0.3–0.7]	0.2 [0.1–0.3]
		
***Causes of mortality***	***(n = 120)***	***(n = 160)***
Violence-related	35 (29.2%) [21.6–37.8]	38 (23%) [16.9–30.5]
Non violence-related	85 (70.8%) [62.2–78.4]	122 (77%) [69.5–83.1]

*95%CI

Violence was reported as the main cause of adult mortality in both locations, accounting for almost a third (29.2%) of deaths in Cité Soleil and almost a quarter (23%) of deaths in Martissant. The majority of violence-related deaths were from gunshot wounds (32/35 in Cité Soleil and 28/38 in Martissant). For children <5 years, infectious diseases were the main cause of mortality. Only one instance of violence-related death was reported among children (in Martissant).

The homicide rate for the period under study reached 457/100,000/year in Cité Soleil (95% CI 417–500) and 237/100,000/year in Martissant (95% CI 206–273). Men were predominantly affected, accounting for two-thirds of violence-related deaths in Cité Soleil and nine-tenths in Martissant. This was highest among men aged 15–39, among whom violence-related deaths accounted for over 1,000 violent deaths/100,000 inhabitants/year in Cité Soleil (95% CI: 1045–1175) and 600 violent deaths/100,000 inhabitants/year in Martissant (95% CI: 577–675).

### Interpersonal violence

In Cité Soleil 22.9% of families (216/945) reported at least one victim of violence. Among these, 91.8% reported one victim, 7.7% reported 2 victims, and 0.5% reported 3 victims within the family. A total of 274 people were victims of violent events, representing 6% of the overall sample; among these 35 died. 81.6% of victims still alive at the time of the survey (195/239) stated that they had suffered direct medical consequences following a violent event, most commonly pain (40%) wounds (24.6%) and fractures (4.6%). A quarter of people (24.6%) reported psychological distress, the main symptoms being stress (30), fear (7), anxiety (4) and worry (2).

In Martissant, 18.6% of families (335/1,800) reported at least one victim of violence, again the majority (93%) reporting a single victim. Overall, when those who had died from violence-related events were included (38), 392 people – 4% of the overall sample size – were found to be victims of violence. Over two-thirds (70.8%) of victims still alive at the time of the survey (250/353; 1 missing data) reported having suffered physically as a result of the violence, with pain (39.6%) and wounds (9.6%) most frequently reported. Psychological distress was also common, affecting 38% of respondents. The main symptoms reported were trauma (23), shock (19), fear (17) and stress (15) (Table 2).

**Table 2:** Main medical consequences of violence

	**Cité Soleil (n = 195)** **n (%)**	**Martissant (n = 250)** **n (%)**
Pain	78 (40%)	99 (39.6%)
Wounds	48(24.6%)	24 (9.6%)
Psychological distress	48 (24.6%)	95 (38%)

NB: respondents could give more than one answer

74.1% (177/239) of people directly affected by violence in Cité Soleil stated they were still affected by the consequences of the violence at the time of the survey, either physically (87, 49.2%) or emotionally (93, 52.5%). Similarly in Martissant, 68.3% (235/344 – 10 missing data) of victims of violence said they were still affected emotionally (143, 61.1%) and physically (50, 21.3%).

### Displacement

Violence can result in substantial population displacement, which may be permanent or temporary. For the first survey that was carried out, in Cité Soleil, we considered only permanent displacement by asking surveyors to count all abandoned households (12% of visited houses). This was in keeping with the findings of the pilot survey. However, during the survey we learnt that temporary displacement was also common. Therefore, for the Martissant survey we included a question on temporary displacement, which revealed that 36.0% of families (648/1,800) had been displaced at least once since January 2006. Temporary displacement was considerably higher amongst those families who were victims of violence (50.3%) than those who were not (30.6%).

### Loss/destruction of property/possessions

Violence resulted in considerable damage to property and other belongings. In Cité Soleil over half of families (450, 52.4%) reported damage to property or belongings, while in Martissant 14.9% (268) reported at least one instance of such damage (Table 3). Overall, in Cité Soleil, of the total sample, some 27.0% of families (255/945) had their house shot at and 19.9% of families (188/945) were victims of theft.

**Table 3:** Destruction of property/possessions

	**Cité Soleil (n = 450)** **n (%)**	**Martissant (n = 268)** **n (%)**
House targeted/hit by gunshot	255 (56.7%)	41 (15.4%)
Theft of belongings	188 (41.8%)	137 (51.3%)
House destroyed/burnt down	38 (8.4%)	32 (12%)
Destruction of belongings	35 (7.8%)	61 (22.8%)

NB: respondents could give more than one answer

### Access to health care following a violent event

At the same time as health care needs increased due to the violence, access to health services was limited by insecurity and poverty. Our survey found that 11% (22/196) of victims of violence in Cité Soleil and 23% (49/212) in Martissant were unable to access care due to insecurity or lack of money. In both settings, around 40% of victims of violence sought care via the informal health sector (such as traditional healers). In Cité Soleil, recourse to the informal sector after a violent event was higher for victims living further away from the hospital (47.3% of people seeking care via the informal sector compared to those living in the sub-districts surrounding the hospital (25%)). This is likely in part due to the limited movement due to insecurity.

## Comment

Previous surveys have reported on the impact of violence during the most violent period of conflict (2004–2005) immediately following the departure of president Aristide [2]. Our survey findings show that the population continued to be affected by high levels of violence for at least another year. In Cité Soleil, the most affected district, we found mortality rates beyond emergency thresholds for the region, and this excess mortality is attributable to violence.

Both crude mortality (0.4 deaths/10,000 people/day) and <5 mortality (0.5 deaths/10,000/day) in Cité Soleil were below mortality rates reported in most conflict settings [5] but nevertheless were beyond the level of emergency thresholds for mortality in Latin America (0.3/10,000/day for adults; 0.4/10,000/day for children <5) [6]. While mortality data are often used to determine the severity of a humanitarian crisis, it has been suggested that mortality thresholds should not be the only indicators to define emergency humanitarian situations, and that other data such as magnitude of displacement, deteriorating security, and targeting of civilians should also be taken into account [5]. Moreover, data on morbidity and coverage of interventions against the main known risk factors for poor health outcomes (in this instance access to health care post-violence) have been argued to be more useful indicators for targeting relief programmes [7]. Taken together, our findings on mortality, violence, and access to care present an alarming situation. In both Cité Soleil and Martissant, MSF started intervening before mortality rates were known on the basis of high incidence of violence and limited access to healthcare for the enclaved populations.

The homicide rate for the period under study reached 457/100,000/year in Cité Soleil and 237/100,000/year in Martissant. These rates are very high compared to data reported from other Latin American contexts, ranging from 6.4/100,000/day (Buenos Aires, Argentina) to 248/100,000/day (Medellin, Colombia) [8]. These rates, if extended to the population of these two areas for the period surveyed, would represent 1,400 violence-related deaths in Cité Soleil and more than 600 in Martissant. This would therefore add another 2,000 deaths to the estimated 8,000 deaths that occurred during the period following the departure of president Jean-Bertrand Aristide up until end of 2005 in the greater Port-au-Prince area [2]. Moreover, it is known that a number of other poorer districts (such as Carrefour, Bel-Air, Cité De Dieu, La Saline) were also severely affected by violence during this period, although no data are available for these districts to our knowledge. One surprising finding was the low <5 mortality rate in Martissant. Based on discussions with the survey teams, the most likely explanation is that households sent their children to live outside the zones of conflict.

In addition to high mortality, the impact of violence on morbidity was substantial, as observed in other settings [9], while at the same time access to essential health services was limited by insecurity or cost. Given the relatively high level of recourse to informal health services, estimations of levels of violence based on official (health-facility based) statistic alone risks considerably underestimating the reality of the violence in this context.

### Limitations

There are several limitations to our survey. First, the survey only covered two districts and results cannot be extrapolated to other areas. Second, as a cross-sectional, retrospective recall survey, the results are subject to several biases. Recall bias is a problem in all self-reporting surveys, and it was not possible to cross-reference individual reports with clinic records or death certificates. The use of self-reporting questionnaires can also lead to certain forms of violence being underestimated. Notably, sexual and domestic violence were rarely reported in comparison to other surveys in Haiti that found high levels of sexual violence [2]. Third, cluster sampling is less precise and more prone to bias [7], although the large number of clusters used (200) would minimize bias by allowing for greater between-cluster variation [10]. Finally, it is conceivable that those families who had fled the area would have been affected by the violence, leading to an underestimation of the effects of violence in our survey.

Nevertheless, these limitations would likely lead to an underestimation of the effects of violence and do not negate the overall conclusion of unacceptable levels of violence being inflicted upon a population that was already marginalized and extremely poor.

## Conclusion

Humanitarian agencies are increasingly responding to situations of urban violence, given the substantial impact on civilian mortality and morbidity, as highlighted by this survey. Further reflection on the best way to organize effective humanitarian assistance in these settings is warranted to ensure that victims of violence can access care even in contexts of high insecurity.

The humanitarian consequences of urban violence are similar to those of armed conflict: people are killed, injured and displaced; infrastructure is damaged or destroyed; access to health care is restricted. In Cité Soleil and Martissant, civilians were exposed to violence in ways that allowed everyone to become a victim; such a situation is comparable to contexts of civil war where the line between combatant and non-combatant is often blurred. Settings of urban violence often comprise a diverse number of armed actors operating within a limited geographical space, and this presents a considerable challenge for negotiating access and obtaining security guarantees for international humanitarian aid agencies. At the same time, international humanitarian law, to which aid agencies appeal to gain access to civilians, may not apply.

From our experience of working in Port au Prince, two measures emerge as particularly important. First, points of evacuation should be negotiated with all fighting parties so that emergency cases can be transferred out of the zone of violence. Second, policy measures are needed to ensure that essential health services are provided free of charge in situations of violence so that victims of violence, who may also have lost all means of financial security, are not excluded from care. In this setting, emergency case management of victims of violence needed to be complemented by work to re-establish essential health services, including psychosocial care, in an area that had been neglected by the formal health sector for years due to high levels of insecurity.

Although the situation in terms of security has improved today, the population affected by violence remains extremely vulnerable and in need of additional humanitarian assistance to meet their basic health needs.

## Competing interests

The authors declare that they have no competing interests.

## Authors' contributions

FP and MvH designed and co-ordinated the study. NF provided the conceptual framing of the findings and wrote the first draft of the paper, and led subsequent drafts. MvH oversaw the implementation of the survey while FP and SM managed data collection in the field. CB provided statistical support for the design and analysis. All authors contributed to the final writing of the paper.
